# ECP versus ruxolitinib in steroid-refractory acute GVHD – a retrospective study by the EBMT transplant complications working party

**DOI:** 10.3389/fimmu.2023.1283034

**Published:** 2023-12-11

**Authors:** Olaf Penack, Christophe Peczynski, William Boreland, Jessica Lemaitre, Ksenia Afanasyeva, Brian Kornblit, Manuel Jurado, Carmen Martinez, Annalisa Natale, Jose Antonio Pérez-Simón, Lucia Brunello, Daniele Avenoso, Stefan Klein, Zubeyde Nur Ozkurt, Concha Herrera, Stina Wichert, Patrizia Chiusolo, Eleni Gavriilaki, Grzegorz W. Basak, Hélène Schoemans, Christian Koenecke, Ivan Moiseev, Zinaida Peric

**Affiliations:** ^1^ Medical Clinic, Department for Haematology, Oncology and Tumorimmunology, Charité Universitätsmedizin Berlin, Berlin, Germany; ^2^ EBMT Transplant Complications Working Party, Paris, France; ^3^ EBMT Paris Study Office, Department of Haematology, Saint Antoine Hospital, INSERM UMR-S 938, Sorbonne University, Paris, France; ^4^ Department of Haematology, First Pavlov State Medical University of St. Petersburg, St. Petersburg, Russia; ^5^ Department of Haematology, Rigshospitalet, Copenhagen, Denmark; ^6^ Department of Haematology, Hospital Universitario Virgen de las Nieves, Granada, Spain; ^7^ Department of Hematology, Hospital Clínic of Barcelona, IDIBAPS, University of Barcelona, Barcelona, Spain; ^8^ Department of Haematology, Ospedale Civile, Pescara, Italy; ^9^ Department of Hematology, University Hospital Virgen del Rocio, Instituto de Biomedicina de Sevilla (IBIS)/CSIC, Universidad de Sevilla, Sevilla, Spain; ^10^ Department of Haematology, SS. Antonio e Biagio e C. Arrigo, Alessandria, Italy; ^11^ Department of Haematology, Kings’ College Hospital, London, United Kingdom; ^12^ Department of Haematology, Universitaetsmedizin, Mannheim, Germany; ^13^ Department of Haematology, Gazi University Faculty of Medicine, Ankara, Türkiye; ^14^ Servicio de Hematología Hospital Universitario Reina Sofía, IMIBIC, University of Cordoba, Cordoba, Spain; ^15^ Department of Haematology, Skane University Hospital, Lund, Sweden; ^16^ Fondazione Policlinico Universitario A. Gemelli IRCCS, Università Cattolica del Sacro Cuore-Roma, Rome, Italy; ^17^ Department of Haematology, Papanicolaou G. Hospital, Thessaloniki, Greece; ^18^ Department of Hematology, Oncology and Internal Medicine, The Medical University of Warsaw, Warsaw, Poland; ^19^ Department of Hematology, University Hospitals Leuven and KU Leuven, Leuven, Belgium; ^20^ Department of Hematology, Hemostasis, Oncology and Stem Cell Transplantation, Hannover Medical School, Hannover, Germany; ^21^ Department of Haematology, University Hospital Centre Zagreb, Zagreb, Croatia

**Keywords:** ECP, GvHD, ruxolinitib, steroid-refractory, allogeneic stem cell transplantation

## Abstract

**Introduction:**

Extracorporal Photophoresis (ECP) is in clinical use for steroid-refractory and steroid-dependent acute GVHD (SR-aGVHD). Based on recent Phase-III study results, ruxolitinib has become the new standard of care for SR-aGVHD. Our aim was to collect comparative data between ruxolitinib and ECP in SR-aGVHD in order to improve the evidence base for clinical decision making.

**Methods:**

We asked EBMT centers if they were willing to participate in this study by completing a data form (Med-C) with detailed information on GVHD grading, -therapy, -dosing, -response and complications for each included patient.

**Results:**

31 centers responded positively (14%) and we included all patients receiving alloSCT between 1/2017-7/2019 and treated with ECP or ruxolitinib for SR-aGVHD grades II-IV from these centers. We identified 53 and 40 patients with grades II-IV SR-aGVHD who were treated with ECP and ruxolitinib, respectively. We performed multivariate analyses adjusted on grading and type of SR-aGVHD (steroid dependent vs. refractory). At day+90 after initiation of treatment for SR-aGVHD we found no statistically significant differences in overall response. The odds ratio in the ruxolitinib group to achieve overall response vs. the ECP group was 1.13 (95% CI = [0.41; 3.22], p = 0.81). In line, we detected no statistically significant differences in overall survival, progression-free survival, non-relapse mortality and relapse incidence.

**Discussion:**

The clinical significance is limited by the retrospective study design and the current data can’t replace prospective studies on ECP in SR-aGVHD. However, the present results contribute to the accumulating evidence on ECP as an effective treatment option in SR-aGVHD.

## Background

Allogeneic stem cell transplantation (alloSCT) is a standard procedure for the treatment of hematologic diseases and other illnesses. The use of alloSCT is constantly increasing with nearly 20.000 transplantations reported to the European Society for Blood and Marrow Transplantation (EBMT) per year (1). AlloSCT has cured many patients with life threatening diseases since its broader use in clinical medicine about forty years ago. However, alloSCT has the downside of causing considerable treatment-related mortality, mainly driven by graft-versus-host disease (GVHD). Acute GVHD (aGVHD) remains one of the major concerns. In a recent EBMT analysis, approximately 30% of all alloSCT recipients had overall grade II-IV aGVHD, which usually requires treatment with high dosed steroids (e.g. 2mg/kg prednisolone or methylprednisolone) (2). In case the treatment with steroids is not successful, the term steroid-refractory aGVHD (SR-aGVHD) is used. There is no published high-quality data from larger mulitcenter patient populations on the incidence and outcome of SR-aGVHD. However, approximatly one third of patients with aGVHD treated with steriods become refractory and have increased non-relapse mortality (3, 4). Patients with SR-aGVHD have typicallly a high mortality despite effective novel drugs, such as ruxolitinib, and there is urgent medical need to develop new strategies (5).

Extracorporal photopheresis (ECP) has been successfully used for treatment of SR-aGVHD (6–8). During ECP autologous mononuclear peripheral blood cells are collected by apheresis and exposed to ultraviolet A light as well as 8methoxypsoralen. ECP offers either an alternative or an add-on to standard immunosuppression and may be associated with less toxicities and side effects, but there is no convincing published evidence from comparable trials as proof of a better toxicity profile. Another possible benefit that has suggested for ECP is the steroid sparing effect, when combined with steroid treatment (7).

Due to absence of a general availability of ECP and also reflecting the lack of randomized trials comparing ECPs efficacy and toxicity with newer treatment options including ruxolitinib, there is a high variety in between treatment centers regarding their use of ECP. While studies have shown the effectiveness and safety of ECP in SR-aGVHD treatment, there is limited data to show how it is being used in the real world setting since ruxolitinib became available.

In the current study we used the EBMT database to retrospectively study treatment patterns and outcomes of SR-aGVHD treatment with ECP versus ruxolitinib. Our aim was to improve the evidence basis on the potential benefit of ECP use as treatment of SR-aGVHD in the current treatment landscape.

## Methods

This is a retrospective multicentre analysis using the data set of the EBMT registry. The EBMT is a voluntary working group of more than 600 transplant centres that are required to report regular follow up on all consecutive stem cell transplantations. Audits are routinely performed to determine the accuracy of the data. The study was planned and approved by the Transplant Complications Working Party of the EBMT. All patients gave their written informed consent to use their personal information for research purposes. The study was conducted in accordance with the Declaration of Helsinki and Good Clinical Practice guidelines.

We first identified all patients in the registry who had a grade II-IV aGvHD and invited these 227 centers to report if their patients had received ECP/Ruxo treatment for SR aGvHD. We then asked centers if they were willing to participate in this study by completing a data form (Med-C, **Supplementary Data**) with very detailed information on GVHD grading, -therapy, -dosing, -response and complications for each included patient. 31 centers responded positively (14%) and we included all patients receiving alloSCT between 1/2017-7/2019 and treated with ECP or Ruxolitinib for SR-aGVHD grades II-IV from these centers.

Inclusion criteria were:

1. AlloSCT recipients in the EBMT database who developed SR-aGVHD after first alloSCT on or after Jan 1st, 2017 but before July 1st, 2019.
2. Patients who initiated treatment with ECP or ruxolitinib within 60 days of onset of SR-aGvHD.
3. Overall grade: II-IV aGVHD only at time of treatment initiation of either ECP or ruxolitinib.
4. Patients who are ≥ 18 years at time of treatment initiation.

Exclusion criteria were:

1. Patients on a clinical trial for GVHD in the retrospective period.
2. Patients who received ECP or ruxolitinib before the onset of steroid-refractory acute GvHD.

Data collected included recipient and donor characteristics (age, sex, cytomegalovirus serostatus and Karnofsky performance status score), diagnosis and status at transplant, interval from diagnosis to transplant, and transplant-related factors, including conditioning regimen, use of anti-thymocyte globulin or Alemtuzumab for pre-transplant *in vivo* T- cell depletion, stem cell source, *ex-vivo* T-cell depletion and post-transplant GVHD prophylaxis. Grading of acute GvHD was performed using established criteria (9). For the purpose of this study, all necessary data were collected according to the EBMT guidelines, using the EBMT Minimum Essential Data forms as well as Med-C forms (see **Supplementary Data**).

### Statistical analysis

The primary endpoint was overall response rate (ORR) at 90 days after initiation of treatment. Secondary endpoints comprised classical survival outcomes: Overall Survival (OS), Progression-Free Survival (PFS), Relapse Incidence (RI) and Non-Relapse-Mortality (NRM), as well as incidence of infectious complications. Start time was the date of start of ECP or ruxolitinib for all endpoints.

ORR at 90 days was defined as being in complete or partial response to the treatment 90 days after introduction of treatment. Death before 90 days was considered as a failure of the treatment. NRM was defined as death without relapse/progression and PFS was defined as survival without relapse or progression.

Multivariate logistic regression models were used to evaluate ORR and results were given as odd ratios. OS and PFS were calculated using the Kaplan-Meier method. Cumulative incidence functions were used to estimate RI and NRM in a competing risk setting, death and relapse competing with each other (10). For the estimation of the cumulative incidence of infectious complications, relapse and death were considered to be competing events. Multivariate analyses were performed using the Cox proportional-hazards model for all survival endpoints. All tests were 2-sided. Statistical analyses were performed with R 4.1.2 software (R Development Core Team, Vienna, Austria) packages.

## Results

### Patient- and transplantation characteristics

We identified 53 and 40 patients with grades II-IV SR-aGVHD who were treated with ECP or ruxolitinib, respectively between January 1st, 2017 and July 1st, 2019 in the EBMT database. Major patient- disease- and transplant characteristics were evenly distributed between the groups (**Table 1**). Patients were transplanted for Acute Leukemia (53.8%), MDS/MPN (25.8%), Chronic Leukemia (11.8%) or Lymphoma (8.6%). Stem cell donors were unrelated (65.4%), identical siblings (18.3%) or haploidentical (18.3%). Patient median age was 52.5 years, with a majority of female recipients (52.7%) and male donors (65.6%). Anti-T-cell globulin (ATG, also termed anti-thymocyte globulin) was given in 39.6%. GVHD prophylaxis was calcineurin inhibitor + methotrexate in 40.7%, calcineurin inhibitor + mycophenolate mofetil in 23.1% and post transplantation cyclophosphamide based in 27.5%.

**Table 1:** Characteristics of both cohorts.

Variable	Level	ECP (n=53)	Ruxo (n=40)	Overall (n=93)	P-value
Year of transplantation	median (min-max) [IQR]	2018 (2016-2019) [2017-2018]	2017 (2016-2019) [2017-2018]	2018 (2016-2019) [2017-2018]	0.39
Cell source	Bone marrow	10 (18.9%)	8 (20%)	18 (19.4%)	0.89
	Peripheral blood	43 (81.1%)	32 (80%)	75 (80.6%)	
Type of donor	Matched related	12 (23.7%)	6 (15%)	18 (19.4%)	Not done
	MUD 10/10	16 (30.2%)	22 (55%)	38 (40.9%)	
	mMUD 9/10	9 (17%)	5 (12.5%)	14 (15.1%)	
	mMUD 8/10 or less	1 (1.9%)	2 (5%)	3 (3.2%)	
	UD (unknown mismatch)	2 (3.8%)	1 (2.5%)	3 (3.2%)	
Diagnosis	Acute leukaemia	30 (56.6%)	20 (50%)	50 (53.8%)	Not done
	Chronic leukaemia	4 (7.5%)	7 (17.5%)	11 (11.8%)	
	Lymphoma	5 (9.4%)	3 (7.5%)	8 (8.6%)	
	Myelodysplastic/ Myeloproliferative	14 (26.4%)	10 (25%)	24 (25.8%)	
Complete remission at transplant	CR	32 (61.5%)	19 (47.5%)	51 (55.4%)	0.18
	No CR	20 (38.5%)	21 (52.5%)	41 (44.6%)	
	missing	1	0	1	
Patient age (years)	median (min-max) [IQR]	52.6 (18.1-73.8) [42.4-60.6]	51.4 (20.2-69.9) [36.5-60.9]	52.5 (18.1-73.8) [39.8-60.8]	0.62
Patient sex	Male	27 (50.9%)	17 (42.5%)	44 (47.3%)	0.42
	Female	26 (49.1%)	23 (57.5%)	49 (52.7%)	
Karnofsky score	>= 90	36 (67.9%)	30 (75%)	66 (71%)	0.46
	< 90	17 (32.1%)	10 (25%)	27 (29%)	
HCT-CI Sorror score	0	25 (49%)	23 (59%)	48 (53.3%)	0.53
	1 or 2	12 (23.5%)	9 (23.1%)	21 (23.3%)	
	3+	14 (27.5%)	7 (17.9%)	21 (23.3%)	
	missing	2	1	3	
Intensity of conditioning	RIC	21 (41.2%)	20 (50%)	41 (45.1%)	0.40
	MAC	30 (58.8%)	20 (50%)	50 (54.9%)	
	missing	2	0	2	
TBI	No	45 (84.9%)	33 (82.5%)	78 (83.9%)	0.75
	Yes	8 (15.1%)	7 (17.5%)	15 (16.1%)	
*In vivo* T-cell depletion	ATG/Campath	19 (37.3%)	17 (42.5%)	36 (39.6%)	0.61
	No	32 (62.7%)	23 (57.5%)	55 (60.4%)	
	missing	2	0	2	
Type of GVHD prophylaxis	CNI + MTX	20 (39.2%)	17 (42.5%)	37 (40.7%)	Not done
	CNI + MMF	14 (27.5%)	7 (17,5%)	21 (23.1%)	
	PTCY based	13 (25.5%)	12 (30%)	25 (27.5%)	
	Sirolimus based	3 (5.9%)	2 (5%)	5 (5.5%)	
	Other	1 (2%)	2 (5%)	3 (3.3%)	
	Missing	2	0	2	

ATG, anti-T-cell globulin; CNI, calcineurin inhibitors; HCT-CI, hematopoietic cell transplantation comorbidity index; MMF, mycophenolate mofetil; MUD, matched unrelated donor; MTX, methotrexate; TBI, total body irradiation.

The median follow-up time was 43.7 months [95% CI 40.1-50.1] in the ECP group and 42.3 months [95% CI 23.8-45.4] in the ruxolitinib group.

### Characteristics of SR-aGVHD

SR-aGVHD is described in **Table 2**. The majority of patients (57%) had been treated with additional drugs/strategies for SR-aGVHD before ECP or ruxolitinib was started. These included most frequently calcineurin inhibitors and mycophenolate mofetil, but also etanercept, mesenchymal stroma cells, methotrexate and fecal microbiota transplantation have been used. The median treatment duration of ECP was 64 days (min-max; 6-1150) [IQR 28.2-150.2] and of ruxolitinib 66.5 days median (min-max; 4-807) [IQR 22.5-207.5].

**Table 2:** Characteristics of acute GVHD.

Variable	Level	ECP (n=53)	Ruxo (n=40)	Overall (n=93)	P-value
Type of steroid	Prednisone	25 (47.1%)	13 (32.5%)	36 (38.7%)	Not done
	Methylprednisone	28 (42.9%)	29 (67.5%)	57 (61.3%)	
Steroid initial dose (mg/kg/day)	median (min-max) [IQR]	2 (0.5-2.5) [1-2]	1 (0.3-2) [1-2]	2 (0.3-2.5) [1-2]	Not done
	missing	1	0	1	
Time between start and end of steroids (days)	median (min-max) [IQR]	88 (3-1213) [50.2-171.8]	49.5 (3-293) [18-109.5]	75.5 (3-1213) [35-137.8]	Not done
	missing	9	2	11	
Other systemic drugs or strategies used to treat aGvHD (other than steroids)	No other drugs/strategies	23 (43.4%)	17 (42.5%)	40 (43%)	Not done
	CNI	22 (41.5%)	15 (37.5%)	37 (39.8%)	
	MMF	5 (9.4%)	4 (10%)	9 (9.7%)	
	Sirolimus	5 (9.4%)	5 (12,5%)	10 (10.8%)	
	Others #	5 (9.4%)	5 (12,5%)	10 (10.8%)	
Time start steroids to SR onset (days)	median (min-max) [IQR]	15 (0-157) [9-40]	12.5 (3-91) [7-21.2]	13 (0-157) [8-25]	Not done
Time from GVHD Diagnosis to first treatment with ECP or Ruxo (days)	median (min-max) [IQR]	29 (7-185) [14-73]	21.5 (5-113) [14-41.3]	23 (1-185) [13-49.5]	Not done
Time from steroid refractory diagnosis to first treatment with ECP or Ruxo (days)	median (min-max) [IQR]	5 (0-38) [3-13]	0 (0-51) [0-4.5]	3 (0-51) [0-11]	Not done
Type of steroid refractory	Steroid-dependant	18 (34%)	3 (7.5%)	21 (22.6%)	0.003
	Steroid-refractory	35 (66%)	37 (92.5%)	72 (77.4%)	
Acute GvHD overall grade (at start of SR treatment)	Grade II	20 (37.7%)	9 (22.5%)	29 (31.2%)	0.13
	Grade III	19 (35.8%)	13 (32.5%)	32 (34.4%)	
	Grade IV	14 (26.4%)	18 (45%)	32 (34.4%)	
Skin aGvHD grade (at start of SR treatment)	0	19 (36.5%)	12 (31.6%)	31 (34.4%)	Not done
	1	4 (7.7%)	3 (7.9%)	7 (7.8%)	
	2	10 (19.2%)	6 (15.8%)	16 (17.8%)	
	3	19 (36.5%)	12 (31.6%)	31 (34.4%)	
	4	0 (0%)	5 (13.2%)	5 (5.6%)	
	missing	1	2	3	
Liver aGvHD grade (at start of SR treatment)	0	34 (66.7%)	23 (59%)	57 (63.3%)	Not done
	1	7 (13.7%)	2 (5.1%)	9 (10%)	
	2	4 (7.8%)	4 (10.3%)	8 (8.9%)	
	3	5 (9.8%)	6 (15.4%)	11 (12.2%)	
	4	1 (2%)	4 (10.3%)	5 (5.6%)	
	missing	2	1	3	
Lower GI aGvHD grade (at start of SR treatment)	0	22 (43.1%)	8 (21.1%)	30 (33.7%)	Not done
	1	4 (7.8%)	3 (7.9%)	7 (7.9%)	
	2	4 (7.8%)	3 (7.9%)	7 (7.9%)	
	3	8 (15.7%)	10 (26.3%)	18 (20.2%)	
	4	13 (25.5%)	14 (36.8%)	27 (30.3%)	
	missing	2	2	4	
Upper GI aGvHD grade (at start of SR treatment)	0	29 (58%)	27 (73%)	56 (64.4%)	Not done
	1	21 (42%)	10 (27%)	31 (35.6%)	
	missing	3	3	6	

CNI, calcineurin inhibitors; MMF, mycophenolate mofeti.

# Others: Etanercept, mesenchymal stroma cells, methotrexate, fecal microbiota transplantation.

Clinically relevant differences were: 1) The ruxolitinib group contained significantly more patients with steroid-refractory aGVHD (92.5%) vs. steroid-dependant aGVHD (7.5%) as compared with the ECP group, where steroid-refractory was 66% vs. 34% steroid-dependant (p=0.003); and 2) we found a tendancy towards a higher proportion of patients with severe overall aGVHD grades III-IV at start of treatment in the Ruxolitinib group (87.5%) vs. the ECP group (62.2%) (p=0.13).

### Key efficacy outcome parameters

The primary outcome parameter in our study was overall response rate (ORR) at day +90 after initiation of ECP or Ruxolitinib. In the ECP group ORR at +90 days was 58.3% (95% CI = [43.2; 72.4]) vs. 47.5% (95% CI = [31.5; 63.9]) in the ruxolitinib group. We next performed multivariate analysis adjusted on aGVHD overall grading (2 vs 3 vs 4) and on the type of aGVHD (steroid-dependent vs. steroid-refractory). We found no statistically significant differences in ORR at day +90 between ECP and ruxolitinib groups (**Table 3**). The odd ratio in the ruxolitinib group to achieve overall response vs. the ECP group was 1.13 (95% CI = [0.41; 3.22], p = 0.81). As expected, acute GVHD overall grades III (OR = 0.33, 95% CI = [0.09; 1.10], p = 0.08) and IV (OR = 0.07, 95% CI = [0.02; 0.26], p < 0.001) were significant risk factors for not achieving an overall response at day +90. In contrast steroid-refractory vs. steroid-dependent GVHD was a non-significant risk factor for not achieving an overall response (OR = 0.44, 95% CI = [0.10; 1.65], p = 0.24).

**Table 3:** Multivarate analyses.

Variable	Hazard ratio/Odd ratio[95% CI]	P
Overall response rate at day +90	1.13 [0.41;3.22]	0.81
Overall survival	0.73 [0.42-1.29]	0.28
Progression-free survival	0.76 [0.43-1.35]	0.35
Relapse incidence	0.95 [0.33-2.77]	0.93
Non-relapse mortality	0.72 [0.36-1.42]	0.34

Results are given for the Ruxolitinib group with the ECP group being the reference.

**Figure 1:**
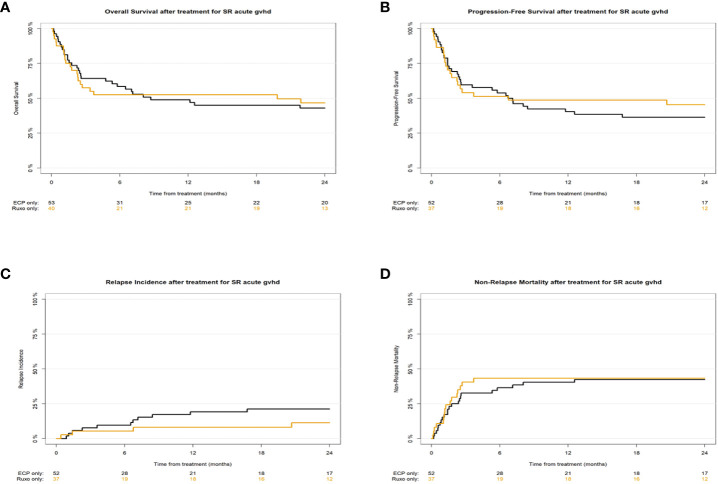
Univariate outcome graphs showing overall survival **(A)**, progression-free survival **(B)**, relapse incidence **(C)** and non-relapse mortality **(D)** in patients with SR-aGVHD after initiation of treatment with ECP (black lines ――) or Ruxolitinib (orange lines ――).

We detected no statistically significant differences in survival or relapse of the underlying malignancy between the two ECP and ruxulitinib cohorts. Univariate outcome graphs are shown in **Figure 1**: overall survival (**Figure 1A**), progression free survival (**Figure 1B**), relapse incidence (**Figure 1C**) and non-relapse mortality (**Figure 1D**). The results of the multivariate analyses are described in **Table 3**. Hazard ratios for the ruxolitinib group with the ECP group being the reference were for overall survival 0.73 [95% CI 0.42-1.29], progression free survival 0.76 [95% CI 0.43-1.35], relapse incidence 0.95 [95% CI 0.33-2.77] and non-relapse mortality 0.72 [95% CI 0.36-1.42].

### Safety- infectious complications

Infections occurred frequently in these high-risk patients with SR-aGVHD. The most common were bacteraemia and viremia with a 1-year incidence of respectively 56.9% (95% CI [42.2-69.2]) and 56.7% (95% CI [42.1-69]) in the ECP group, 48.7% (95% CI [32.1-63.4]) and 55.3% (95% CI [37.8-69.7]) in the Ruxolitinib group. The 1-year incidence of fungemia was 7.5% (95% Ci [2.4-16.7]) in the ECP group and 17.5% (95% CI [7.6-30.8]) in the Ruxolitinib group.

Overall, the most reported infections were bacteremia (49 patients), CMV reactivation (26 pts), pneumonia (17 pts) and invasive fungal disease (11 pts).Less frequent reported infections included upper respiratory tract infections (3 pts), urinary tract infections (8 pts), skin infections (1 pt) and clostridium difficile colitis (3 pts) as well as various less frequent infections (14 pts).

In multivariate analysis adjusted on the grade of acute GVHD and the type of steroid refractory, patients treated with Ruxolitinib were found to develop significantly less bacteraemia infections than patients treated with ECP (HR 0.44, 95% CI [0.21-0.9], p=0.025). No significant difference was observed regarding viremia (HR 0.87, 95% CI [0.44-1.73], p=0.69).

### Patients who received ECP and ruxolitinib

During the study period, we identified additional 30 alloSCT recipients with SR-aGVHD who were treated with a combination of ECP and ruxolitinib. There was variety in treatment durations and treatment sequences. These patients were not included in the current analyses. However, we observed that in some centers combination treatments of ECP and ruxolitinib were already in clinical use during the study period 2017-2019.

## Discussion

In the present retrospective study of SR-aGVHD treatment with ECP versus ruxolitinib, we extensively collected data using specifically designed data sheets (so called Med-C forms) and detected no statistically significant differences in major clinical parameters: overall response rate at day +90 as well as overall survival, progression-free survival, non-relapse mortality and relapse incidence. The clinical significance is limited by the retrospective study design and we need to be cautious with interpretation. For example, we had no information on the total number of prior lines of GVHD therapy in both groups and we do not know why centers decided to use ECP or ruxolitinib as well as steroid dosages in individual patients. On top of this only 14% of centers agreed to participate, due to the extensive workload. We can’t compare the center characteristics of responding centers to non-responding centers. Therefore, our results may not be representative for the entire European transplant cohort. We can’t conclude from these data that ECP is equally efficacious as compared to ruxolitinib in this indication. This question needs to be addressed in prospective studies on ECP in SR-aGVHD. However, our present results add more data to the already accumulating evidence on ECP as an effective treatment option in SR-aGVHD (7, 8, 11, 12).

We found an overall response rate of ECP treatment in SR-aGVHD at day+90 of 58% and in the ruxolitinib arm 47%. The somewhat higher response rate in the ECP arm is probably not a sign of higher efficacy (as shown in multivariate analyses), but most likely explained by differences in patient characteristics: The ruxolitinib group contained significantly more patients with steroid-refractory aGVHD vs. steroid-dependant aGVHD as compared with the ECP group. In multivariate analyses we found that steroid-refractory versus steroid-dependent aGVHD was a risk factor for worse overall response. Therefore, the ruxolitinib-treated group contained more high risk patients. On top of that we found a tendancy towards a higher proportion of patients with severe overall aGVHD grades at start of treatment in the Ruxolitinib group, which also points towards a difficult to treat population. Due to a variety in patient populations and also in SR-aGVHD definitions and treatment-response definition this is hard to compare the response rates observed in our study to results in the literature. However, overall our results in the ECP group are in line with previously published evidence. The previously reported overall response rates of ECP in this indication are heterogeneous. A meta-analysis identified 6 studies with 54 aGVHD patients (also steroid sensitive patients were included) treated with ECP. The pooled proportion of ORR for ECP in various organs was 69% with a high heterogeneity between studies (11). Another study focused exclusively on patients with SR-aGVHD, comparing ECP (n = 57) and anti-cytokine therapy (n = 41) (12). The overall best response rate in the ECP arm was 66% and in the same range like in our study. In multivariate analyses the use of ECP was associated with improved response rates ad compared to the anti-cytokine group. The same is true for the response rates of ruxolitinib treatment in our study versus published evidence: it is not easily comparable but seems to be roughly in a similar range. We found 47.5% overall response rate of SR-aGVHD at day+90, whereas the seminal phase III trial resulted in 62% overall response rate at day +28 of 62% and durable overall responses at day +56 of 40% (13).

The ECP-mediated beneficial effect on GVHD is believed to be rather immunomodulatory than exclusively immunosuppressive. ECP has been demonstrated to induce apoptosis of antigen-reactive immune cells during GVHD and supports a more anti-inflammatory cytokine profile as well as expansion of regulatory T-cells (14). Therefore on potential benefit of ECP could be the lower risk of infectios complications as compared to treatment with immunosuppressive substances, such as ruxolitinib. In the contrary direction, ECP often requires central venous access and this may lead to transmission of skin microbiota to the blood circulation increasing the risk for bacteremia. We therefore were specifiically interested in the patterns and frequencies of common infections complications in patiens with aGVHD. Our finding that the frequency of common infections complications, such as cytomegaly virus (CMV) reactivations, pneumonia and invasive fungal disease was not hugely different in ECP treated vs. ruxolitinib treated patients with SR-aGVHD argue against a pronounced difference in infection risk between the two treatment modalities. Of note, our results and conclusions are limited by the fact that we do not have detailed information on anti-infective prophylaxis regimens that have been used in both arms and may have influenced outcome. Due to the retrospective character of our study and the lack of prophylaxis data, we can’t exclude - or proof - that anti-infective prophylaxis, e.g. anti-fungal drugs, were used less frequently in the ECP arm versus the ruxolitinib arm. However, the higher frequency of bacteremia in the ECP group vs. the ruxolitinib group we found was statistically significant. This is not surprising and is most likely an indirect effect because patients with ECP regularly have a central venous catheter (CVC), which massively increases the risk for bacteremias. Our finding that non-relapse-mortality was not different in between the treatment arms argue against a high clinical significance of these bacteremias. Often CVC-related bacteremias that are reported are by gram positive cocci and it is often not clear if these are contaminations from skin flora or real infections. Typically gram positive cocci lead to less severe infections as compared to gram negative bacteria (15). We have no information of additional parameters, such as lengths of hospital stay or frequency of intensive care ward admissions to further analyze the clinical significance of the observed bacteremias. A further limitation is that we do not have information on other potential side effects of ECP or ruxolitinib, such as cytopenias or organ toxicities.

In conclusion we didn’t find any statistically significant difference in overall response rates and survival in patients with SR-aGVHD treated with ECP or ruxolitinib. The clinical significance is limited by the retrospective study design and the current data can’t replace prospective studies on ECP in SR-aGVHD. However, the present results contribute to the accumulating evidence on ECP as an effective treatment option in SR-aGVHD.

## Data availability statement

The raw data supporting the conclusions of this article will be made available by the corresponding author upon request.

## Ethics statement

The studies involving humans were approved by EBMT Transplant Complications Working Party. The studies were conducted in accordance with the local legislation and institutional requirements. The participants provided their written informed consent to participate in the EBMT data registry.

## Author contributions

OP: Conceptualization, Writing – original draft, Writing – review & editing. CP: Formal analysis, Methodology, Visualization, Writing – review & editing. WB: Data curation, Writing – review & editing. JL: Data curation, Methodology, Writing – review & editing. KA: Investigation, Writing – review & editing. BK: Investigation, Writing – review & editing. MJ: Investigation, Writing – review & editing. CM: Investigation, Writing – review & editing. AN: Investigation, Writing – review & editing. JP-S: Investigation, Writing – review & editing. LB: Investigation, Writing – review & editing. DA: Investigation, Writing – review & editing. SK: Investigation, Writing – review & editing. ZO: Investigation, Writing – review & editing. CH: Investigation, Writing – review & editing. SW: Investigation, Writing – review & editing. PC: Investigation, Writing – review & editing. EG: Investigation, Writing – review & editing. GB: Supervision, Writing – review & editing. HS: Supervision, Writing – review & editing. CK: Supervision, Writing – review & editing. IM: Supervision, Writing – review & editing. ZP: Supervision, Writing – review & editing.
