# Supplementary material for: ECP versus ruxolitinib in steroid-refractory acute GVHD – a retrospective study by the EBMT transplant complications working party
Source: Front Immunol. 2023 Dec 11;14:1283034. doi: 10.3389/fimmu.2023.1283034 (PMC10750400; doi:10.3389/fimmu.2023.1283034)
Supplement: Supplementary file 1 [file DataSheet_1.pdf]

Treatment of aGvHD (before steroid refractory (SR) status):

Steroid details:

**Name of steroid**	**Start date**	**Initial dose** *(mg/kg/day)*	**Stop date**
	_ _ _ _/_ _/_ _		_ _ _ _/_ _/_ _

Were other systemic drugs used to treat aGvHD (other than steroids): ☐ Yes ☐ No

If yes, please list drugs below:

**Name of drug**

Steroid Refractory* aGvHD details:

**Steroid refractory definition covers other subtypes such as dependent and intolerant, but 'steroid refractory' will be used as an umbrella term, as per the protocol*

SR a-GvHD date of onset? _ _ _ _/_ _/_ _

SR a-GvHD type?
☐ *Steroid-refractory*
☐ *Steroid-dependent*

aGvHD grade (at start of SR treatment)? ____________

aGvHD organ involved (at start of SR treatment):

	**Stage of aGvHD** *(using Glucksberg scale)*
Skin	☐ Stage 0<br>☐ Stage I<br>☐ Stage II<br>☐ Stage III<br>☐ Stage IV<br>☐ Not Assessed

Liver	☐ Stage 0<br>☐ Stage I<br>☐ Stage II<br>☐ Stage III<br>☐ Stage IV<br>☐ Not Assessed
Lower GI tract	☐ Stage 0<br>☐ Stage I<br>☐ Stage II<br>☐ Stage III<br>☐ Stage IV<br>☐ Not Assessed
Upper GI tract	☐ Stage 0<br>☐ Stage I<br>☐ Stage II<br>☐ Stage III<br>☐ Stage IV<br>☐ Not Assessed

Treatment of the SR aGvHD

Start date of the SR aGvHD treatment with ECP or Ruxolitinib - if both, whichever came first:

_ _ _ _/ _ _/ _ _
(yyyy/mm/dd)

ECP details:

Was ECP administ ered?	Start date *(yyyy/mm/ dd)*	Initial schedule	**Schedule** *(after start of SR GvHD treatment with ECP)*						Reason for stopping treatment
			**At 28 days** *(or at closest possible date of evaluation)*	**At 56 days** *(or at closest possible date of evaluation)*	**At 90 days** *(or at closest possible date of evaluation)*	**At 180 days** *(if not already covered in cGvHD section)*	**Was ECP stopped for more than 6 weeks and restarted?**	**Is treatment ongoing?**	

☐ Yes<br>☐ No		**A** ☐ *3 treatments per week*<br>**B** ☐ *2 consecutive days per week*<br>**C** ☐ *2 consecutive days every two weeks*<br>**D** ☐ *1 day per week*<br>**E** ☐ *1 day every two weeks*<br>**F** ☐ *1 day per month*<br>**G** ☐ *2 consecutive days per month*<br>☐ *Other:* _ _ _ _ _ _	*Schedule:*<br>☐ **A**<br>☐ **B**<br>☐ **C**<br>☐ **D**<br>☐ **E**<br>☐ **F**<br>☐ **G**<br>☐ Other schedule: _ _ _ _	*Schedule:*<br>☐ **A**<br>☐ **B**<br>☐ **C**<br>☐ **D**<br>☐ **E**<br>☐ **F**<br>☐ **G**<br>☐ Other schedule: _ _ _ _	*Schedule:*<br>☐ **A**<br>☐ **B**<br>☐ **C**<br>☐ **D**<br>☐ **E**<br>☐ **F**<br>☐ **G**<br>☐ Other schedule: _ _ _ _	*Schedule:*<br>☐ **A**<br>☐ **B**<br>☐ **C**<br>☐ **D**<br>☐ **E**<br>☐ **F**<br>☐ **G**<br>☐ Other schedule: _ _ _ _	☐ Yes<br>☐ No<br>*Date stopped*<br>_ _ _ _/_ _/_ _<br>*Date restarted*<br>_ _ _ _/_ _/_ _	☐ Yes<br>☐ No<br>**If no:**<br>*Stop date*<br>_ _ _ _/_ _/_ _	☐ *Responded to treatment*<br>☐ *No response*<br>☐ *Stopped at patient request*<br>☐ *Adverse event*<br>☐ *Patient died*<br>☐ *Insufficient veneous access*<br>☐ *Not stated*<br>☐ *Other:* _ _ _ _
			Date of evaluation: _ _ _ _/_ _/_ _	*Date of evaluation:* _ _ _ _/_ _/_ _	*Date of evaluation:* _ _ _ _/_ _/_ _	*Date of evaluation:* _ _ _ _/_ _/_ _			

Ruxolitinib details:

Was Ruxolitinib administered?	Start date *(yyyy/mm/dd)*	Initial dose	**Dose** *(after start of SR GvHD treatment with Ruxolitinib)*						Reason for stopping treatment
			**At 28 days** *(or at closest possible date of evaluation)*	**At 56 days** *(or at closest possible date of evaluation)*	**At 90 days** *(or at closest possible date of evaluation)*	**At 180 days** *(if not already covered in cGvHD section)*	**Was Ruxolitinib stopped and restarted?**	**Is treatment ongoing?**	
☐ Yes<br>☐ No		☐ 10 mg twice a day<br>☐ 5 mg twice a day<br>☐ 5 mg once a day<br>☐ Other*:* _ _ _ _ _ _	Dose: _ _ _ _ _ _<br>☐ mg once a day<br>☐ mg twice a day	Dose: _ _ _ _ _ _<br>☐ mg once a day<br>☐ mg twice a day	Dose: _ _ _ _ _ _<br>☐ mg once a day<br>☐ mg twice a day	Dose: _ _ _ _ _ _<br>☐ mg once a day<br>☐ mg twice a day	☐ Yes<br>☐ No	☐ Yes<br>☐ No	☐ *Responded to treatment*<br>☐ *No response*<br>☐ *Stopped at patient request*<br>☐ *Adverse event*<br>☐ *Patient died*<br>☐ *Not stated*<br>☐ *Other:* _ _ _ _ _
			Date of evaluation: _ _ _ _/_ _/_ _	*Date of evaluation:* _ _ _ _/_ _/_ _	*Date of evaluation:* _ _ _ _/_ _/_ _	*Date of evaluation:* _ _ _ _/_ _/_ _	*Date stopped:* _ _ _ _/_ _/_ _<br>*Date restarted:* _ _ _ _/_ _/_ _	**If no:**<br>*Stop date* _ _ _ _/_ _/_ _	

Steroid details:
(please note that time intervals are different for steroids than for ECP/Ruxolitinib)

Name of steroid	Start date *(yyyy/mm/dd)*	Dose at SR onset *(mg/kg/day)*	**Steroid dose** *(after start of SR GvHD treatment with ECP or Ruxolitinib - whichever comes first)*				Were steroids stopped and restarted?	Is treatment ongoing?
			**At 28 days** *(or at closest possible date of evaluation)*	**At 56 days** *(or at closest possible date of evaluation)*	**At 90 days** *(or at closest possible date of evaluation)*	**At 180 days** *(if not already covered in cGvHD section)*		
			Dose: ______ *(mg/kg/day)*	Dose: ______ *(mg/kg/day)*	Dose: ______ *(mg/kg/day)*	Dose: ______ *(mg/kg/day)*	☐ Yes ☐ No	☐ Yes ☐ No
			Date of evaluation: ____/__/__	*Date of evaluation:* ____/__/__	*Date of evaluation:* ____/__/__	*Date of evaluation:* ____/__/__	*Date stopped:* ____/__/__ *Date restarted:* ____/__/__	**If no:** *Stop date* ____/__/__

Were other systemic therapies used to treat SR aGvHD (other than steroids or Ruxolitinib): ☐ **Yes** ☐ **No**
This includes any treatments that were given before Ruxolitinib or ECP

If yes, please list drugs below:

Name of drug	Start date	Stop date
	____/__/__	____/__/__
	____/__/__	____/__/__
	____/__/__	____/__/__
	____/__/__	____/__/__
	____/__/__	____/__/__
	____/__/__	____/__/__

Response to the treatment of the SR aGvHD

Overall Response

Last recorded response to treatment: **Date of assessment:** ____/__/__

- ☐ *Complete response*
- ☐ *Partial response*

☐ *Stable disease*
☐ *Progressive disease*

Organ specific response (if available):

Organ	**Stage and Grade of aGvHD** *(after start of SR GvHD treatment with ECP or Ruxolitinib - whichever comes first)*				Date of resolution *(yyyy/mm/dd)*
	**At 28 days** *(or at closest possible date of evaluation)*	**At 56 days** *(or at closest possible date of evaluation)*	**At 90 days** *(or at closest possible date of evaluation)*	**At 180 days** *(or at closest possible date of evaluation)*	
	Date of evaluation: _ _ _ _/_ _/_ _	*Date of evaluation:* _ _ _ _/_ _/_ _	*Date of evaluation:* _ _ _ _/_ _/_ _	*Date of evaluation:* _ _ _ _/_ _/_ _	
Skin	☐ Stage 0<br>☐ Stage I<br>☐ Stage II<br>☐ Stage III<br>☐ Stage IV<br>☐ Not Assessed	☐ Stage 0<br>☐ Stage I<br>☐ Stage II<br>☐ Stage III<br>☐ Stage IV<br>☐ Not Assessed	☐ Stage 0<br>☐ Stage I<br>☐ Stage II<br>☐ Stage III<br>☐ Stage IV<br>☐ Not Assessed	☐ Stage 0<br>☐ Stage I<br>☐ Stage II<br>☐ Stage III<br>☐ Stage IV<br>☐ Not Assessed	_ _ _ _/_ _/_ _
Liver	☐ Stage 0<br>☐ Stage I<br>☐ Stage II<br>☐ Stage III<br>☐ Stage IV<br>☐ Not Assessed	☐ Stage 0<br>☐ Stage I<br>☐ Stage II<br>☐ Stage III<br>☐ Stage IV<br>☐ Not Assessed	☐ Stage 0<br>☐ Stage I<br>☐ Stage II<br>☐ Stage III<br>☐ Stage IV<br>☐ Not Assessed	☐ Stage 0<br>☐ Stage I<br>☐ Stage II<br>☐ Stage III<br>☐ Stage IV<br>☐ Not Assessed	_ _ _ _/_ _/_ _
Lower GI tract	☐ Stage 0<br>☐ Stage I<br>☐ Stage II<br>☐ Stage III<br>☐ Stage IV<br>☐ Not Assessed	☐ Stage 0<br>☐ Stage I<br>☐ Stage II<br>☐ Stage III<br>☐ Stage IV<br>☐ Not Assessed	☐ Stage 0<br>☐ Stage I<br>☐ Stage II<br>☐ Stage III<br>☐ Stage IV<br>☐ Not Assessed	☐ Stage 0<br>☐ Stage I<br>☐ Stage II<br>☐ Stage III<br>☐ Stage IV<br>☐ Not Assessed	_ _ _ _/_ _/_ _
Upper GI tract	☐ Stage 0<br>☐ Stage I<br>☐ Stage II<br>☐ Stage III<br>☐ Stage IV<br>☐ Not Assessed	☐ Stage 0<br>☐ Stage I<br>☐ Stage II<br>☐ Stage III<br>☐ Stage IV<br>☐ Not Assessed	☐ Stage 0<br>☐ Stage I<br>☐ Stage II<br>☐ Stage III<br>☐ Stage IV<br>☐ Not Assessed	☐ Stage 0<br>☐ Stage I<br>☐ Stage II<br>☐ Stage III<br>☐ Stage IV<br>☐ Not Assessed	_ _ _ _/_ _/_ _
Overall grade of aGvHD	☐ Grade 0<br>☐ Grade I<br>☐ Grade II<br>☐ Grade III<br>☐ Grade IV<br>☐ Not Assessed	☐ Grade 0<br>☐ Grade I<br>☐ Grade II<br>☐ Grade III<br>☐ Grade IV<br>☐ Not Assessed	☐ Grade 0<br>☐ Grade I<br>☐ Grade II<br>☐ Grade III<br>☐ Grade IV<br>☐ Not Assessed	☐ Grade 0<br>☐ Grade I<br>☐ Grade II<br>☐ Grade III<br>☐ Grade IV<br>☐ Not Assessed	_ _ _ _/_ _/_ _

Overall response<br>*(if organ specific staging is not available)*	☐ *Complete response*<br>☐ *Partial response*<br>☐ *Stable disease*<br>☐ *Progressive disease*<br>☐ Not Assessed	☐ *Complete response*<br>☐ *Partial response*<br>☐ *Stable disease*<br>☐ *Progressive disease*<br>☐ Not Assessed	☐ *Complete response*<br>☐ *Partial response*<br>☐ *Stable disease*<br>☐ *Progressive disease*<br>☐ Not Assessed	☐ *Complete response*<br>☐ *Partial response*<br>☐ *Stable disease*<br>☐ *Progressive disease*<br>☐ Not Assessed	_ _ _ _/_ _ /_ _
